# Potential Therapeutic Features of Human Amniotic Mesenchymal Stem Cells in Multiple Sclerosis: Immunomodulation, Inflammation Suppression, Angiogenesis Promotion, Oxidative Stress Inhibition, Neurogenesis Induction, MMPs Regulation, and Remyelination Stimulation

**DOI:** 10.3389/fimmu.2019.00238

**Published:** 2019-02-20

**Authors:** Mohsen Abbasi-Kangevari, Seyyed-Hadi Ghamari, Fahimeh Safaeinejad, Soheyl Bahrami, Hassan Niknejad

**Affiliations:** ^1^Department of Pharmacology, School of Medicine, Shahid Beheshti University of Medical Sciences, Tehran, Iran; ^2^Student Research Committee, Social Determinants of Health Center, Shahid Beheshti University of Medical Sciences, Tehran, Iran; ^3^Ludwig Boltzmann Institute for Experimental and Clinical Traumatology in AUVA Research Center, Vienna, Austria

**Keywords:** angiogenesis, anti-inflammation, antioxidant, amniotic membrane, mesenchymal stem cell, multiple sclerosis, neurogenesis

## Abstract

Multiple sclerosis (MS) is an inflammatory and degenerative disorder of the central nervous system with unknown etiology. It is accompanied by demyelination of the nerves during immunological processes in the presence of oxidative stress, hypoxia, cerebral hypo-perfusion, and dysregulation in matrix metalloproteinases (MMPs). Human amniotic mesenchymal stem cells (hAMSCs) as pluripotent stem cells possess some conspicuous features which could be of therapeutic value in MS therapy. hAMSCs could mimic the cascade of signals and secrete factors needed for promoting formation of stable neovasculature and angiogenesis. hAMSCs also have immunomodulatory and immunosuppressive effects on inflammatory processes and reduce the activity of inflammatory cells, migration of microglia and inhibit recruitment of certain immune cells to injury sites. hAMSCs attenuate the oxidative stress supported by the increased level of antioxidant enzymes and the decreased level of lipid peroxidation products. Furthermore, hAMSCs enhance neuroprotection and neurogenesis in brain injuries by inhibition of inflammation and promotion of neurogenesis. hAMSCs could significantly increase the expression of neurotrophic factors, which prevents neurons from initiating programmed cell death and improves survival, development, and function of neurons. In addition, they induce differentiation of neural progenitor cells to neurons. hAMSCs could also inhibit MMPs dysregulation and consequently promote the survival of endothelial cells, angiogenesis and the stabilization of vascular networks. Considering the mentioned evidences, we hypothesized here that hAMSCs and their conditioned medium could be of therapeutic value in MS therapy due to their unique properties, including immunomodulation and inflammation suppression; angiogenesis promotion; oxidative stress inhibition; neurogenesis induction and neuroprotection; matrix metalloproteinases regulation; and remyelination stimulation.

## Introduction

Multiple sclerosis (MS) is an inflammatory disorder of the central nervous system which is accompanied by neural demyelination, axonal loss, and disability. Although the main etiology of MS is unknown, genetic, environmental and infectious agents may be among the factors that play a role in the development of MS (1). The main pathologic processes of MS include inflammatory and degenerative phases (2). The presence of inflammatory cells and their secreted molecules in the demyelinating lesions supports the notion that pathogenic T cells that react with myelin antigens play a key role in MS, which then results in the degeneration of neurons (3). In addition, the migration of autoreactive T cells to the central nervous system results in destroying the central neurons and their myelin sheaths, which is mediated by matrix metalloproteinases (MMPs) (4). Furthermore, oxidative stress, hypoxia and cerebral hypo-perfusion may lead to increased demyelinating lesions in MS (5, 6).

Although various pharmacological and non-pharmacological therapeutic approaches have been used in the treatment of MS, no definite cure of MS has been discovered to date. Moreover, all the approved therapeutic approaches are expected to be life-long, while their potential adverse effects may compromise their safety or patients' adherence to treatment. Therefore, it is imperative yet challenging to find new safe therapies with fewer delivery concerns and less adverse effects that are more effective in slowing or preventing MS progression, have the potential to reverse patients' disability, and consequently improve patients' adherence.

Cell-based therapy by mesenchymal stem cells (MSCs) is one of the therapeutic approaches that has drawn attention as a potential approach to address these challenges, considering initial promising research results (7). Nevertheless, there are uncertainties regarding the use of MSCs in MS treatment including tissue source, route of delivery, cell number, dosing, adverse effects, safety monitoring, and duration of action (8).

Human amniotic membrane is considered a potential source for MSCs which possesses unique biological properties, including anti-inflammatory, anti-fibrosis, anti-scarring and low immunogenicity characteristics (9, 10). Human amniotic mesenchymal stem cells (hAMSCs) are pluripotent stem cells that could be isolated from the fibroblastic layer of human amniotic membrane.

Altogether, it seems that hAMSCs have potential therapeutic features that could be promising in MS therapy. There is some evidence for some hAMSCs properties including immunomodulation and inflammation suppression (11); angiogenesis promotion (12, 13); oxidative stress inhibition (14); neurogenesis induction and neuroprotection (15); matrix metalloproteinase regulation (16); and remyelination stimulation.

## Supporting Evidence for Hypothesis

### Immunomodulation and Inflammation Suppression

Inflammation in the central nervous system is one of the major pathogenic processes in MS. Although the trigger of the inflammatory response in MS is still not clear, it is suggested that MS is developed when auto-reactive T cells target proteins which exist predominantly in myelin and on axons (17, 18). Besides T cells, B cells, macrophages, activated microglia, and dendritic cells act as key players in the pathogenesis of multiple sclerosis (19). When dendritic cells are exposed to myelin-derived antigens, they secrete cytokines which induce the differentiation of naive T cells into effector T cells in secondary lymphoid tissues (2). Inflammatory cells migrate across the blood-brain-barrier to the central nervous system and thus cause the inflammatory lesions which are characterized by an area of demyelination of nerves and axonal loss. In addition, poor T-regulatory functioning enhances the expansion of inflammatory responses (20).

Efficient treatment of MS depends on developing a therapeutic method that can specifically target and regulate immune responses. A number of immunomodulatory or immunosuppressive drugs including Interferon-β, Glatiramer acetate, Natalizumab, and Fingolimod have been designed to target the immune component of MS. Although these drugs have displayed beneficial effects for halting MS, they have shown little impact on its progression (21). On the other hand, mesenchymal stem cells could be applied as therapeutics in MS through imposing their immunomodulatory effect by inducing a shift in T cells from a pro-inflammatory to an anti-inflammatory state (22); inhibiting naive and memory T cell proliferation and maturation (23); inhibiting proliferation, secretion of proinflammatory cytokines, and cytotoxicity of natural killer cells and natural killer T cells; inhibiting B cell proliferation, and antibodies production; inhibiting the initial differentiation of monocytes to dendritic cells and impairing their activation, and antigen presentation (24); and inhibiting the chemotactic activity of neutrophils (25). In addition, they promote the generation of regulatory T cells (23), preserve neutrophil viability and function (26), and regulate macrophage recruitment (27) and function (28).

hAMSCs could lead to a decrease of peripheral blood mononuclear cells, interferon-gamma and interleukin-17 production (29). They might also reduce migration, recruitment, and activity of a broad range of immune cells, including T cells, natural killer cells, natural killer T cells, dendritic cells, B cells, neutrophils, monocytes, and macrophages at injury sites (30). The secretion of nitric oxide by hAMSCs could be the main cause of their immunosuppressive effect (11). Currently, several soluble factors, either produced constitutively by hAMSCs or as a result of cross-talk with target immune cells, have been shown to exert the immunomodulatory properties of MSCs, including indoleamine2,3- dioxygenase, prostaglandin E2, interleukin-10, interleukin-6, HLA-G, transforming growth factor-b1, and hepatocyte growth factor (23, 31–33). In addition, hAMSCs have shown a positive effect on immunosuppression in mice models of MS (2, 18).

### Angiogenesis Promotion

Cerebral hypoperfusion as well as vascular factors are involved in neurovascular dysfunction, vascular oxidative stress, and relative tissue hypoxia, which could increase the risk of developing demyelinating lesions as observed in MS. Therefore, cerebral hypoperfusion may represent pathologic factors or neuroprotective processes involved in recovery or progression of MS (6).

Although various therapeutic approaches have been utilized to promote angiogenesis, most approaches still cannot fully mimic the process of natural vessel development. The use of hAMSCs has been explored to mimic the cascade of signals needed for enhancing viability and promoting formation of stable neovasculature (16). We also showed the inducing effects of hAMSCs conditioned media on the sprouting of endothelial cells (12, 13). MSCs' potential for angiogenesis relies on their ability to differentiate to smooth muscle cells and endothelial cells as well as their paracrine effects by angiogenetic factors. Angiogenic factors secreted by MSCs vary based on the source and include VEGF, bFGF, MCP-1, SDF-1, angiopoietin, monocyte chemoattractant protein, interleukin-6, placental growth factor, and cysteine-rich angiogenic inducer 61, which could regulate vascular network remodeling (34). Moreover, hAMSCs could promote angiogenesis by inducing the extracellular signaling-regulated kinase 1/2-MAPK signaling pathway (35). Therefore, conditioned medium from hAMSCs could be beneficial for promoting angiogenesis and would probably enhance tissue repair.

### Oxidative Stress Inhibition

The inflammatory processes play a significant role in neural tissue injury (36) and in MS pathogenesis critically involve Reactive Oxygen Species (ROS). Infiltrated immune cells, macrophages and activated microglia could generate immense amounts of oxidizing radicals including superoxide, hydrogen peroxide and nitric oxide. In addition, the activation of immature myeloid cells induce synthesis of nitric oxide and reactive oxygen species (5). Free radicals can also activate nuclear transcription factor-kappa B (NF-κB). NF-κB upregulates the expression of genes involved in MS, including tumor necrosis factor-α (TNF-α), nitric oxide synthase (iNOS), intracellular adhesion molecule 1 (ICAM-1) and vascular-cell adhesion molecule 1 (VCAM-1) (37).

Treatment of mice model of MS with antioxidant enzymes markedly suppressed the severity of MS (38). In addition, mitochondrial stabilization and ROS-mediated phagocytosis of myelin may reduce axonal damage in mice models of MS (39–41).

The utilization of MSCs in mice models of MS can inhibit the production of inflammatory factors, including nitric oxide (NO), tumor necrosis factor, IL1-β and reactive oxygen species by activated microglia and preventing neuronal damage. The intravenous injection of MSCs showed a string antioxidant effect in a mice model of MS through the high expression of antioxidant enzymes including catalase, superoxide desmutase and poly (ADP-ribose) polymerase-1 during MSC treatment (42).

In addition, hAMSCs transplantation into transgenic mice increased the level of antioxidant enzymes and decreased the level of lipid peroxidation and oxidative stress (14).

### Neurogenesis Induction, Neuroprotection, and Remyelination Stimulation

Neurodegeneration is considered as a major contributor to neurological disability in MS and might be the dominant underlying process of progressive MS. Whether the neurodegeneration is an independent process or due to inflammatory processes remains unknown (43). There are studies that report the mechanisms of neurodegeneration in MS, including the accumulation of amyloid precursor protein in neurons and a reduction in the N-acetyl-aspartate/Creatine ratio (2). In addition, damage to mitochondrial DNA and mitochondrial enzyme complexes may lead to neurodegeneration (44). Axonal density reduction in the white matter and spinal cords of MS patients is another probable mechanism of neurodegeneration (45).

Another process involved in pathogenesis of MS is demyelination. Demyelination causes myelin-producing oligodendrocytes to undergo apoptosis and thus results in myelin loss (46). In response to demyelination, activated resident oligodendrocyte progenitor cells proliferate, migrate to affected areas, and differentiate to replace lost oligodendrocytes, which might lead to myelin reconstitution and functional recovery (47, 48). However, remyelination is typically incomplete or defective and many lesions remain demyelinated (48, 49). This could be either due to the limited ability of mature oligodendrocytes to compensate for myelin loss (50) or to the failure of oligodendrocyte progenitor cells to successfully generate new myelinating cells (48). Therefore, oligodendrocyte progenitor cells cannot often compensate myelin loss on their own.

Treatment of MS with hAMSCs could significantly increase the expression of neurotrophic factors including NGF, CNTF, and BDNF (51). Neurotrophins prevent neurons from initiating programmed cell death and improve survival, development, and function of neurons (15, 52, 53). In a study, hAMSCs promoted neurological recovery in rats after intracranial hemorrhage. It was concluded that the mechanism of action was mediated by inhibition of inflammation and apoptosis, increasing neurotrophic factors expression, and promoting neurogenesis and angiogenesis (54). hAMSCs induce differentiation of progenitor cells to neurons (52). In addition, hAMSCs have the ability to differentiate neural and glial cells in response to induction medium (55). The level of neurotrophins significantly decreases in the CNS of MS patients and is correlated with neuron damage. Therefore, increasing the levels of neurotrophins—or at least maintaining their physiological levels—in MS patients might be of therapeutic value (56, 57). The therapeutic effects of hAMSCs were able to improve the motor functions of neurodegenerative diseases in mice models significantly (58), which indicates that hAMSCs have the potential to differentiate into neural cells. Therefore, hAMSCs could potentially promote neurogenesis, neuroprotection, and remyelination.

### Matrix Metalloproteinase Regulation

MMPs are a family of a large number of proteolytic enzymes that have received much attention in neuro-inflammatory diseases. Leukocyte infiltration through the blood-brain-barrier is dependent on several factors including secretion of tumor necrotizing factor-α, gelatinase B/MMP-9 and gelatinase A/MMP-2 (59). It has been shown that the expression of MMPs leads to degradation of extracellular matrix proteins of the basal lamina which surrounds blood vessels (60). MMP-9 is a major matrix metalloproteinase in the pathogenesis of multiple sclerosis and experimental autoimmune encephalitis, which could enhance leukocyte migration, blood-brain-barrier disruption and myelin lysis (4). Different attempts have been made to develop inhibitors of MMPs for the potential treatment of diseases in which MMPs play a major role. In one study, treatment of MS patients with Natalizumab decreased the risk of progressive multifocal encephalopathy by inhibition of MMP-9 (61).

Mesenchymal stem cells application in inflammatory diseases resulted in decreased levels of MMPs or reduced MMPs activity. In another study, a conditioned medium of MSCs decreased disease severity by inhibition of the MMPs activity rate in inflammatory arthritis (62). In another study, MSCs were introduced as robust sources of MMPs inhibition that were mediated by Tissue Inhibitors of Metalloproteinase (TIMPs). This may have therapeutic effects in inflammatory and vascular diseases (63). It has been shown that treatment with mesenchymal stem cells inhibited dysregulation of both MMPs and TIMPs after focal ischemic stroke, which facilitated neurological and functional recovery after stroke (64).

## Hypothesis

**Figure 1:**
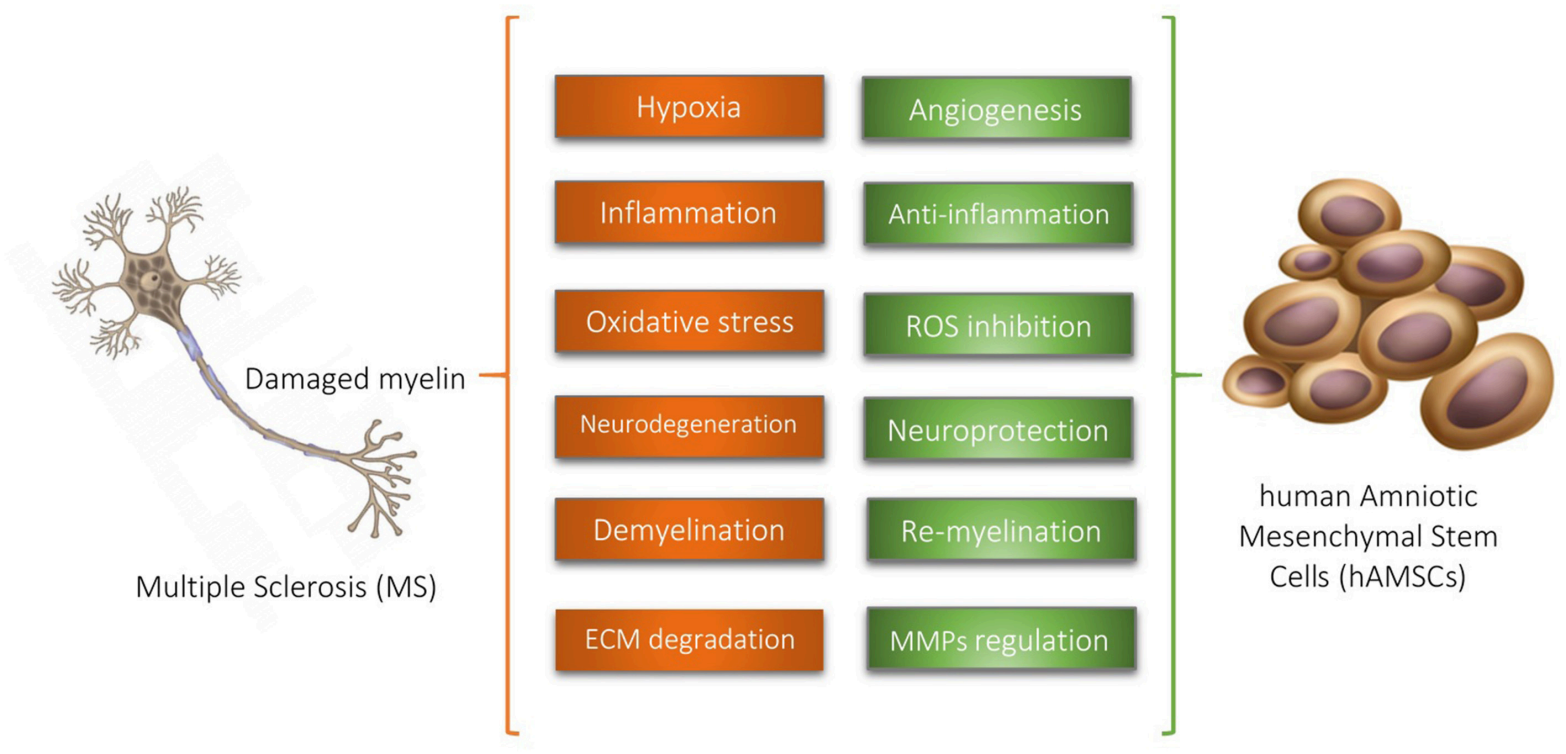
Human Amniotic Mesenchymal Stem Cells (hAMSCs) have potential therapeutic features in multiple sclerosis via supporting evidences illustrated above. ROS, Reactive Oxidative Species; ECM, Extracellular Matrix; MMPs, Matrix Metalloproteinases.

Considering the supporting evidences, we hypothesize that hAMSCs have potential therapeutic features in multiple sclerosis via angiogenesis promotion, inflammation suppression, oxidative stress inhibition, neurogenesis induction, neuroprotection, MMPs regulation, and remyelination stimulation (Figure 1).

### Evaluation of Hypothesis

The hypothesis will be evaluated by conducting *in vitro* and *in vivo* experimental studies to address the building block questions of the hypothesis.

#### *In vitro* Studies

To evaluate the *in vitro* effects of hAMSCs conditioned medium, oligodendrocyte cell lines such as the OLN-93 will be cultured. To determine neuroprotection and oxidation inhibition of hAMSCs conditioned medium, first oxidative stress and cell death will be induced by using H_2_O_2_ or cuprizone. Afterwards, viability, qualitative and quantitative levels of apoptotic markers as well as neurotrophin levels will be measured and mitochondrial assays will be conducted before and after treatment with hAMSCs conditioned medium. The concentration-dependence to exert the immunomodulatory actions of hAMSCs will be investigated by mixed lymphocyte reaction assay.

#### *In vivo* Studies

To evaluate the *in vivo* effects of hAMSCs conditioned medium, the toxic model of multiple sclerosis will be created in C57BL/6 mice by cuprizone. To measure the motor coordination and balance among mice, rotarod performance testing will be carried out before and after treatment with hAMSCs conditioned medium. To determine the effect of hAMSCs conditioned medium on angiogenesis, VEGF levels will be evaluated by ELISA kit. To determine inflammation suppression by hAMSCs conditioned medium, inflammatory and anti-inflammatory cytokines will be measured before and after treatment with hAMSCs conditioned medium by ELISA kits.

To evaluate direct injection of hAMSCs, the cells would be locally injected in the affected regions of the brain tissue. In addition, as MSCs express a variety of chemokine receptors including CXCR4 and CCR2, and cell adhesion molecules including CD44, integrins α4 and β1, and CD99 (65), they could be delivered via intravenous administration. Therefore, we aim to compare the effectiveness of the ways of administration of hAMSCs in MS models in future studies. In addition, to evaluate the effect of the number of cells administered in future studies, 1 × 10^6^ hAMSCs (66) will be injected into the tail vein of rat and the number of cells will be adjusted if necessary. Results will be evaluated after 6 weeks for investigation of the acute phase of inflammation and 12 weeks to evaluate the chronic phase of the MS model.

To evaluate oxidative stress inhibition by hAMSCs, the reactive oxygen species and glutathione reduction is measured before and after treatment with hAMSCs conditioned medium by glutathione assay kit.

To evaluate the effects of hAMSCs conditioned media on OPC differentiation for neurogenesis and remyelination, oligodendrocyte precursor marker, Olig2, and adult myelin markers, PLP, MBP, MOG, will be measured by real-time PCR and Western blot analyses. To further investigate the effects of hAMSCs, cellular and molecular tests using immunohistochemistry, Western blot and real time-PCR analyses on the myelin genes and proteins as well as microscopic examinations will be carried out. Finally, to determine the effect of hAMSCs conditioned medium on MMPs regulation, MMPs activity will be determined by MMPs activity assay.

## Discussion

hAMSCs have unique properties including inflammation suppression, angiogenesis promotion, oxidative stress inhibition, neurogenesis induction, neuroprotection, MMPs regulation, and remyelination stimulation, which are of potential therapeutic value for MS therapy. The anti-inflammatory feature of hAMSCs, along with their ability to promote neurogenesis and differentiation of progenitor cells to neurons, could make them valuable sources for prevention and therapy in both early and advanced stages of MS. hAMSCs possess other conspicuous characteristics to make them practical sources for cell-based therapies against MS, which are discussed further here in brief. The amniotic membrane, which will be obtained from elective Cesarean sections to eliminate contaminations, is an attractive source of MSCs since large quantities of hAMSCs could be cost-effectively collected without invasive procedures and ethical concerns (67). Although hAMSCs are capable of self-renewal and differentiation to all the three embryonic layers, they are not tumorigenic and thus could be considered safe and easy-to-access sources for cell-based MS therapy (68). As we have shown previously, cryopreservation could be used as a viable system for banking human amniotic cells with low cost in terms of expense, time and personnel involved and its ease of implementation. Therefore, they are suitable for banking and establishing ready-to-use sources for cell therapy (13). Since hAMSCs retain their reproducible biologic characteristics, they can be sufficiently expanded for use in regenerative medicine (69). In addition, the differentiation of hAMSCs to different cell lines relies on growth factors provided in the medium which can be altered in the laboratory. Amniotic stem cells can differentiate to neuron and glial cells (55); therefore, by regulating the growth factors, hAMSCs could potentially be applied in various neurological disorders. Neurological disorders occur among the elderly, and MSCs are affected by the aging process as indicated by the decrease in the bone marrow MSC pool and also reduced capacity to handle oxidative stress (70). Therefore, allograft transplantation of hAMSCs and the ability to provide large quantities of hAMSCs might make them the choice to substitute allogenic source of MSCs among the elderly. hAMSCs have low immunogenicity, which makes them suitable for transplantation (70). hAMSCs have the ability to pass the blood-brain-barrier and enter the central nervous system (71). Thus, they can be administered intravenously and there is no need for more invasive routes of administration. In addition, intravenous administration of hAMSCs enhances their global access to sites of inflammation and damage to secrete the required factors to alleviate MS considering that MS lesions are disseminated in space, meaning that patients have lesions in several areas of the CNS (72). A question that needs to be clarified is whether hAMSCs give the same effect regardless the degree of MS. The pathogenesis of MS is seemingly different in the early and late stages of the disease. There is less inflammation in the brain in primary progressive MS than in secondary progressive MS. In addition, increased blood T-cell reactivity to myelin sheaths was found in relapsing–remitting MS and secondary progressive MS (73). Therefore, anti-inflammatory and immunomodulatory qualities of hAMSCs could be effective in secondary progressive and relapsing–remitting MS. Acute axonal injury occurs focally in secondary progressive MS and diffusely in primary progressive MS (73). Therefore, neurogenesis, neuroprotection, and remyelination imposed by hAMSCs could be of value via systemic administration in primary progressive MS and via local injection in secondary progressive MS. Taken together, the anti-inflammatory feature of hAMSCs, along with their ability to promote neurogenesis and differentiation of progenitor cells to neurons, could make them valuable sources for prevention and therapy in both early and advanced stages of MS.

To the best of our knowledge, no studies have focused on the unique properties of hAMSCs for MS therapy. Therefore, it is essential to investigate the potential therapeutic values of hAMSCs for MS.

## Conclusion

hAMSCs and their conditioned medium could be of therapeutic value in MS therapy due to their unique properties including immunomodulation and inflammation suppression; angiogenesis promotion; oxidative stress inhibition; neurogenesis induction and neuroprotection; MMPs regulation; and remyelination stimulation. Therefore, it is required to evaluate the hypothesis in future *in vitro* and *in vivo* studies.

## Author Contributions

MA-K, S-HG, FS, and HN hypothesized the idea, wrote the manuscript and revised it. SB revised the manuscript.

### Conflict of Interest Statement

The authors declare that the research was conducted in the absence of any commercial or financial relationships that could be construed as a potential conflict of interest.

## References

[1] Loma I Heyman R. Multiple sclerosis: pathogenesis and treatment. Curr Neuropharmacol. (2011) 9:409–16. 10.2174/157015911796557911 22379455 PMC3151595

[2] Al Jumah MA Abumaree MH. The immunomodulatory and neuroprotective effects of mesenchymal stem cells (MSCs) in experimental autoimmune encephalomyelitis (EAE): a model of multiple sclerosis (MS). Int J Mol Sci. (2012) 13:9298–331. 10.3390/ijms13079298 22942767 PMC3430298

[3] Compston A Coles A. Multiple sclerosis. Lancet (2008) 372:1502–17. 10.1016/S0140-6736(08)61620-7 18970977

[4] Zhang Y Dong H Seeburg DP Wojtkiewicz GR Waterman P Pulli B. Multimodal molecular imaging demonstrates myeloperoxidase regulation of matrix metalloproteinase activity in neuroinflammation. Mol Neurobiol. (2018). [Epub ahead of print]. 10.1007/s12035-018-1137-2 29808380 PMC6261713

[5] Ohl K Tenbrock K Kipp M. Oxidative stress in multiple sclerosis: central and peripheral mode of action. Exp Neurol. (2016) 277:58–67. 10.1016/j.expneurol.2015.11.010 26626971 PMC7094520

[6] Monti L Morbidelli L Rossi A. Impaired cerebral perfusion in multiple sclerosis: relevance of endothelial factors. Biomark Insights (2018) 13:1177271918774800. 10.1177/1177271918774800 29795976 PMC5960845

[7] Sarkar P Rice CM Scolding NJ. Cell therapy for multiple sclerosis. CNS Drugs (2017) 31:453–69. 10.1007/s40263-017-0429-9 28397112

[8] Scolding NJ Pasquini M Reingold SC Cohen JA. Cell-based therapeutic strategies for multiple sclerosis. Brain (2017) 140:2776–96. 10.1093/brain/awx154 29053779 PMC5841198

[9] Niknejad H Peirovi H Jorjani M Ahmadiani A Ghanavi J Seifalian AM. Properties of the amniotic membrane for potential use in tissue engineering. Eur Cell Mater. (2008) 15:88–99. 10.22203/eCM.v015a07 18446690

[10] Tehrani FA Modaresifar K Azizian S Niknejad H. Induction of antimicrobial peptides secretion by IL-1beta enhances human amniotic membrane for regenerative medicine. Sci Rep. (2017) 7:17022. 10.1038/s41598-017-17210-7 29208979 PMC5717175

[11] Yan K Zhang R Chen L Chen F Liu Y Peng L. Nitric oxide-mediated immunosuppressive effect of human amniotic membrane-derived mesenchymal stem cells on the viability and migration of microglia. Brain Res. (2014) 1590:1–9. 10.1016/j.brainres.2014.05.041 24909791

[12] Niknejad H Paeini-Vayghan G Tehrani FA Khayat-Khoei M Peirovi H. Side dependent effects of the human amnion on angiogenesis. Placenta (2013) 34:340–5. 10.1016/j.placenta.2013.02.001 23465536

[13] Yazdanpanah G Paeini-Vayghan G Asadi S Niknejad H. The effects of cryopreservation on angiogenesis modulation activity of human amniotic membrane. Cryobiology (2015) 71:413–8. 10.1016/j.cryobiol.2015.09.008 26432457

[14] Jiao H Shi K Zhang W Yang L Yang L Guan F. Therapeutic potential of human amniotic membrane-derived mesenchymal stem cells in APP transgenic mice. Oncol Lett. (2016) 12:1877–83. 10.3892/ol.2016.4857 27588134 PMC4998013

[15] Kim EY Lee KB Kim MK. The potential of mesenchymal stem cells derived from amniotic membrane and amniotic fluid for neuronal regenerative therapy. BMB Rep. (2014) 47:135–40. 10.5483/BMBRep.2014.47.3.289 24499672 PMC4163884

[16] Jiang F Ma J Liang Y Niu Y Chen N Shen M. Amniotic mesenchymal stem cells can enhance angiogenic capacity via MMPs *in vitro* and *in vivo*. Biomed Res Int. (2015) 2015:324014. 10.1155/2015/324014 26491665 PMC4600487

[17] Lopez-Diego RS Weiner HL. Novel therapeutic strategies for multiple sclerosis–a multifaceted adversary. Nat Rev Drug Discov. (2008) 7:909–25. 10.1038/nrd2358 18974749

[18] McDonald CA Payne NL Sun G Moussa L Siatskas C Lim R. Immunosuppressive potential of human amnion epithelial cells in the treatment of experimental autoimmune encephalomyelitis. J Neuroinflammation (2015) 12:112. 10.1186/s12974-015-0322-8 26036872 PMC4457975

[19] Hoglund RA Maghazachi AA. Multiple sclerosis and the role of immune cells. World J Exp Med. (2014) 4:27–37. 10.5493/wjem.v4.i3.27 25254187 PMC4172701

[20] Danikowski KM Jayaraman S Prabhakar BS. Regulatory T cells in multiple sclerosis and myasthenia gravis. J Neuroinflammation (2017) 14:117. 10.1186/s12974-017-0892-8 28599652 PMC5466736

[21] Payne N Siatskas C Bernard CC. The promise of stem cell and regenerative therapies for multiple sclerosis. J Autoimmun. (2008) 31:288–94. 10.1016/j.jaut.2008.04.002 18504116

[22] Prasanna SJ Gopalakrishnan D Shankar SR Vasandan AB. Pro-inflammatory cytokines, IFNgamma and TNFalpha, influence immune properties of human bone marrow and Wharton jelly mesenchymal stem cells differentially. PLoS ONE (2010) 5:e9016. 10.1371/journal.pone.0009016 20126406 PMC2814860

[23] Magatti M Vertua E Cargnoni A Silini A Parolini O. The immunomodulatory properties of amniotic cells: the two sides of the coin. Cell Transplant. (2018) 27:31–44. 10.1177/0963689717742819 29562786 PMC6434482

[24] Chiesa S Morbelli S Morando S Massollo M Marini C Bertoni A. Mesenchymal stem cells impair *in vivo* T-cell priming by dendritic cells. Proc Natl Acad Sci USA. (2011) 108:17384–9. 10.1073/pnas.1103650108 21960443 PMC3198360

[25] Magana-Guerrero FS Dominguez-Lopez A Martinez-Aboytes P Buentello-Volante B Garfias Y. Human amniotic membrane mesenchymal stem cells inhibit neutrophil extracellular traps through TSG-6. Sci Rep. (2017) 7:12426. 10.1038/s41598-017-10962-2 28963485 PMC5622031

[26] Maqbool M Vidyadaran S George E Ramasamy R. Human mesenchymal stem cells protect neutrophils from serum-deprived cell death. Cell Biol Int. (2011) 35:1247–51. 10.1042/CBI20110070 21649586

[27] Magatti M Vertua E De Munari S Caro M Caruso M Silini A. Human amnion favours tissue repair by inducing the M1-to-M2 switch and enhancing M2 macrophage features. J Tissue Eng Regen Med. (2017) 11:2895–911. 10.1002/term.2193 27396853 PMC5697700

[28] Kim KS Kim HS Park JM Kim HW Park MK Lee HS. Long-term immunomodulatory effect of amniotic stem cells in an Alzheimer's disease model. Neurobiol Aging (2013) 34:2408–20. 10.1016/j.neurobiolaging.2013.03.029 23623603

[29] Kang JW Koo HC Hwang SY Kang SK Ra JC Lee MH. Immunomodulatory effects of human amniotic membrane-derived mesenchymal stem cells. J Vet Sci. (2012) 13:23–31. 10.4142/jvs.2012.13.1.23 22437532 PMC3317453

[30] Wang M Yuan Q Xie L. Mesenchymal stem cell-based immunomodulation: properties and clinical application. Stem Cells Int. (2018) 2018:3057624. 10.1155/2018/3057624 30013600 PMC6022321

[31] Shi Y Su J Roberts AI Shou P Rabson AB Ren G. How mesenchymal stem cells interact with tissue immune responses. Trends Immunol. (2012) 33:136–43. 10.1016/j.it.2011.11.004 22227317 PMC3412175

[32] Meesuk L Tantrawatpan C Kheolamai P Manochantr S. The immunosuppressive capacity of human mesenchymal stromal cells derived from amnion and bone marrow. Biochem Biophys Rep. (2016) 8:34–40. 10.1016/j.bbrep.2016.07.019 28955939 PMC5613701

[33] Dabrowski FA Burdzinska A Kulesza A Sladowska A Zolocinska A Gala K. Comparison of the paracrine activity of mesenchymal stem cells derived from human umbilical cord, amniotic membrane and adipose tissue. J Obstet Gynaecol Res. (2017) 43:1758–68. 10.1111/jog.13432 28707770

[34] Gu W Hong X Potter C Qu A Xu Q. Mesenchymal stem cells and vascular regeneration. Microcirculation (2017) 24. 10.1111/micc.12324 27681821

[35] Wang Y Chen X Yin Y Li S. Human amnion-derived mesenchymal stem cells induced osteogenesis and angiogenesis in human adipose-derived stem cells via ERK1/2 MAPK signaling pathway. BMB Rep. (2018) 51:194–9. 10.5483/BMBRep.2018.51.4.005 29429450 PMC5933215

[36] Khalaj L Peirovi H Khodagholi F Abdi A Dargahi L Ahmadiani A. Acute 17beta-estradiol pretreatment protects against abdominal aortic occlusion induced spinal cord ischemic-reperfusion injury. Neurochem Res. (2011) 36:268–80. 10.1007/s11064-010-0314-0 21080066

[37] Gilgun-Sherki Y Melamed E Offen D. The role of oxidative stress in the pathogenesis of multiple sclerosis: the need for effective antioxidant therapy. J Neurol. (2004) 251:261–8. 10.1007/s00415-004-0348-9 15015004

[38] van Horssen J Witte ME Schreibelt G de Vries HE Radical changes in multiple sclerosis pathogenesis. Biochim Biophys Acta (2011) 1812:141–50. 10.1016/j.bbadis.2010.06.011 20600869

[39] Liu Y Hao W Letiembre M Walter S Kulanga M Neumann H. Suppression of microglial inflammatory activity by myelin phagocytosis: role of p47-PHOX-mediated generation of reactive oxygen species. J Neurosci. (2006) 26:12904–13. 10.1523/JNEUROSCI.2531-06.2006 17167081 PMC6674962

[40] Forte M Gold BG Marracci G Chaudhary P Basso E Johnsen D. Cyclophilin D inactivation protects axons in experimental autoimmune encephalomyelitis, an animal model of multiple sclerosis. Proc Natl Acad Sci USA. (2007) 104:7558–63. 10.1073/pnas.0702228104 17463082 PMC1857227

[41] Ortiz GG Pacheco-Moises FP Bitzer-Quintero OK Ramirez-Anguiano AC Flores-Alvarado LJ Ramirez-Ramirez V. Immunology and oxidative stress in multiple sclerosis: clinical and basic approach. Clin Dev Immunol. (2013) 2013:708659. 10.1155/2013/708659 24174971 PMC3794553

[42] Lanza C Morando S Voci A Canesi L Principato MC Serpero LD. Neuroprotective mesenchymal stem cells are endowed with a potent antioxidant effect *in vivo*. J Neurochem. (2009) 110:1674–84. 10.1111/j.1471-4159.2009.06268.x 19619133

[43] Bermel RA. Unravelling neurodegeneration in multiple sclerosis. Lancet Neurol. (2017) 16:764–6. 10.1016/S1474-4422(17)30302-2 28920874

[44] Su K Bourdette D Forte M. Mitochondrial dysfunction and neurodegeneration in multiple sclerosis. Front Physiol. (2013) 4:169. 10.3389/fphys.2013.00169 23898299 PMC3722885

[45] Lee JY Taghian K Petratos S. Axonal degeneration in multiple sclerosis: can we predict and prevent permanent disability? Acta Neuropathol Commun. (2014) 2:97. 10.1186/s40478-014-0097-7 25159125 PMC4243718

[46] Glenn JD Smith MD Kirby LA Baxi EG Whartenby KA. Disparate Effects of mesenchymal stem cells in experimental autoimmune encephalomyelitis and cuprizone-induced demyelination. PLoS ONE (2015) 10:e0139008. 10.1371/journal.pone.0139008 26407166 PMC4583481

[47] El Waly B Macchi M Cayre M Durbec P Oligodendrogenesis in the normal and pathological central nervous system. Front Neurosci. (2014) 8:145 10.3389/fnins.2014.00145 24971048 PMC4054666

[48] Kremer D Gottle P Hartung HP Kury P. Pushing Forward: Remyelination as the New Frontier in CNS Diseases. Trends Neurosci. (2016) 39:246–63. 10.1016/j.tins.2016.02.004 26964504

[49] Rivera FJ Aigner L. Adult mesenchymal stem cell therapy for myelin repair in multiple sclerosis. Biol Res. (2012) 45:257–68. 10.4067/S0716-97602012000300007 23283435

[50] Pomeroy IM Jordan EK Frank JA Matthews PM Esiri MM. Focal and diffuse cortical degenerative changes in a marmoset model of multiple sclerosis. Mult Scler. (2010) 16:537–48. 10.1177/1352458509360362 20194580 PMC2874633

[51] Shu J He X Li H Liu X Qiu X Zhou T. The beneficial effect of human amnion mesenchymal cells in inhibition of inflammation and induction of neuronal repair in EAE mice. J Immunol Res. (2018) 2018:5083797. 10.1155/2018/5083797 30035132 PMC6035808

[52] Hempstead BL. Dissecting the diverse actions of pro- and mature neurotrophins. Curr Alzheimer Res. (2006) 3:19–24. 10.2174/156720506775697061 16472198

[53] Reichardt LF. Neurotrophin-regulated signalling pathways. Philos Trans R Soc Lond B Biol Sci. (2006) 361:1545–64. 10.1098/rstb.2006.1894 16939974 PMC1664664

[54] Zhou H Zhang H Yan Z Xu R. Transplantation of human amniotic mesenchymal stem cells promotes neurological recovery in an intracerebral hemorrhage rat model. Biochem Biophys Res Commun. (2016) 475:202–8. 10.1016/j.bbrc.2016.05.075 27188654

[55] Sanluis-Verdes A Sanluis-Verdes N Manso-Revilla MJ Castro-Castro AM Pombo-Otero J Fraga-Marino M. Tissue engineering for neurodegenerative diseases using human amniotic membrane and umbilical cord. Cell Tissue Bank (2017) 18:1–15. 10.1007/s10561-016-9595-0 27830445

[56] Chen X Ma L Jiang Y Chen S Zhu C Liu M. Minocycline up-regulates the expression of brain-derived neurotrophic factor and nerve growth factor in experimental autoimmune encephalomyelitis. Eur J Pharmacol. (2012) 686:124–9. 10.1016/j.ejphar.2012.04.043 22575526

[57] Modi KK Sendtner M Pahan K. Up-regulation of ciliary neurotrophic factor in astrocytes by aspirin: implications for remyelination in multiple sclerosis. J Biol Chem. (2013) 288:18533–45. 10.1074/jbc.M112.447268 23653362 PMC3689994

[58] Rennie K Gruslin A Hengstschlager M Pei D Cai J Nikaido T. Applications of amniotic membrane and fluid in stem cell biology and regenerative medicine. Stem Cells Int. (2012) 2012:721538. 10.1155/2012/721538 23093978 PMC3474290

[59] Gerwien H Hermann S Zhang X Korpos E Song J Kopka K. Imaging matrix metalloproteinase activity in multiple sclerosis as a specific marker of leukocyte penetration of the blood-brain barrier. Sci Transl Med. (2016) 8:364ra152. 10.1126/scitranslmed.aaf8020 27831901

[60] Sorokin L. The impact of the extracellular matrix on inflammation. Nat Rev Immunol. (2010) 10:712–23. 10.1038/nri2852 20865019

[61] Fissolo N Pignolet B Matute-Blanch C Trivino JC Miro B Mota M. Matrix metalloproteinase 9 is decreased in natalizumab-treated multiple sclerosis patients at risk for progressive multifocal leukoencephalopathy. Ann Neurol. (2017) 82:186–95. 10.1002/ana.24987 28681388

[62] Kay AG Long G Tyler G Stefan A Broadfoot SJ Piccinini AM. Mesenchymal stem cell-conditioned medium reduces disease severity and immune responses in inflammatory arthritis. Sci Rep. (2017) 7:18019. 10.1038/s41598-017-18144-w 29269885 PMC5740178

[63] Lozito TP Tuan RS. Mesenchymal stem cells inhibit both endogenous and exogenous MMPs via secreted TIMPs. J Cell Physiol. (2011) 226:385–96. 10.1002/jcp.22344 20665704

[64] Chelluboina B Nalamolu KR Mendez GG Klopfenstein JD Pinson DM Wang DZ. Mesenchymal stem cell treatment prevents post-stroke dysregulation of matrix metalloproteinases and tissue inhibitors of metalloproteinases. Cell Physiol Biochem. (2017) 44:1360–9. 10.1159/000485533 29186705

[65] Liu L Eckert MA Riazifar H Kang DK Agalliu D Zhao W. From blood to the brain: can systemically transplanted mesenchymal stem cells cross the blood-brain barrier? Stem Cells Int. (2013) 2013:435093. 10.1155/2013/435093 23997771 PMC3753739

[66] Nessler J Benardais K Gudi V Hoffmann A Salinas Tejedor L Janssen S. Effects of murine and human bone marrow-derived mesenchymal stem cells on cuprizone induced demyelination. PLoS ONE (2013) 8:e69795. 10.1371/journal.pone.0069795 23922802 PMC3724887

[67] Miyamoto S Ohnishi S Onishi R Tsuchiya I Hosono H Katsurada T. Therapeutic effects of human amnion-derived mesenchymal stem cell transplantation and conditioned medium enema in rats with trinitrobenzene sulfonic acid-induced colitis. Am J Transl Res. (2017) 9:940–52. 28386323 PMC5375988

[68] Phermthai T Thongbopit S Pokathikorn P Wichitwiengrat S Julavijitphong S Tirawanchai N. Carcinogenicity, efficiency and biosafety analysis in xeno-free human amniotic stem cells for regenerative medical therapies. Cytotherapy (2017) 19:990–1001. 10.1016/j.jcyt.2017.04.004 28566211

[69] Miranda-Sayago JM Fernandez-Arcas N Benito C Reyes-Engel A Carrera J Alonso A. Lifespan of human amniotic fluid-derived multipotent mesenchymal stromal cells. Cytotherapy (2011) 13:572–81. 10.3109/14653249.2010.547466 21208022

[70] Wang Y Yin Y Jiang F Chen N. Human amnion mesenchymal stem cells promote proliferation and osteogenic differentiation in human bone marrow mesenchymal stem cells. J Mol Histol. (2015) 46:13–20. 10.1007/s10735-014-9600-5 25432786

[71] Smith CL Chaichana KL Lee YM Lin B Stanko KM O'Donnell T. Pre-exposure of human adipose mesenchymal stem cells to soluble factors enhances their homing to brain cancer. Stem Cells Transl Med. (2015) 4:239–51. 10.5966/sctm.2014-0149 25646527 PMC4339851

[72] Hackett C Knight J Mao-Draayer Y. Transplantation of Fas-deficient or wild-type neural stem/progenitor cells (NPCs) is equally efficient in treating experimental autoimmune encephalomyelitis (EAE). Am J Transl Res. (2014) 6:119–28. 24489991 PMC3902222

[73] Pender MP. The pathogenesis of primary progressive multiple sclerosis: antibody-mediated attack and no repair? J Clin Neurosci. (2004) 11:689–92. 10.1016/j.jocn.2003.12.013 15337125

